# Optimization Strategies for Bruch’s Membrane Opening Minimum Rim Area Calculation: Sequential versus Simultaneous Minimization

**DOI:** 10.1038/s41598-017-14284-1

**Published:** 2017-10-24

**Authors:** Philip Enders, Werner Adler, Friederike Schaub, Manuel M. Hermann, Michael Diestelhorst, Thomas Dietlein, Claus Cursiefen, Ludwig M. Heindl

**Affiliations:** 1 Department of Ophthalmology, University Hospital of Cologne, Kerpener Strasse 62, 50924 Cologne, Germany; 2 Department of Medical Informatics, Biometry and Epidemiology, Friedrich-Alexander University Erlangen-Nuremberg, Erlangen, Germany

## Abstract

To compare a simultaneously optimized continuous minimum rim surface parameter between Bruch’s membrane opening (BMO) and the internal limiting membrane to the standard sequential minimization used for calculating the BMO minimum rim area in spectral domain optical coherence tomography (SD-OCT). In this case-control, cross-sectional study, 704 eyes of 445 participants underwent SD-OCT of the optic nerve head (ONH), visual field testing, and clinical examination. Globally and clock-hour sector-wise optimized BMO-based minimum rim area was calculated independently. Outcome parameters included BMO-globally optimized minimum rim area (BMO-gMRA) and sector-wise optimized BMO-minimum rim area (BMO-MRA). BMO area was 1.89 ± 0.05 mm^2^. Mean global BMO-MRA was 0.97 ± 0.34 mm^2^, mean global BMO-gMRA was 1.01 ± 0.36 mm^2^. Both parameters correlated with r = 0.995 (P < 0.001); mean difference was 0.04 mm^2^ (P < 0.001). In all sectors, parameters differed by 3.0–4.2%. In receiver operating characteristics, the calculated area under the curve (AUC) to differentiate glaucoma was 0.873 for BMO-MRA, compared to 0.866 for BMO-gMRA (P = 0.004). Among ONH sectors, the temporal inferior location showed the highest AUC. Optimization strategies to calculate BMO-based minimum rim area led to significantly different results. Imposing an additional adjacency constraint within calculation of BMO-MRA does not improve diagnostic power. Global and temporal inferior BMO-MRA performed best in differentiating glaucoma patients.

## Introduction

The morphometric parameter Bruch’s membrane opening-based minimum rim area (BMO-MRA) was proposed by Gardiner and associates for two-dimensional neuro-retinal rim tissue measurement of the optic nerve head (ONH) using spectral domain optical coherence tomography (SD-OCT)^1^. In addition to the clinically available parameters, Bruch’s membrane opening-based minimum rim width (BMO-MRW) and peripapillary retinal nerve fibre layer (RNFL) thickness, two studies on BMO-MRA showed high diagnostic power for all ONH sizes and equalized differences in BMO-MRW between very large and very small ONHs^1,2^.

SD-OCT of the optic nerve head based on Bruch’s membrane opening has been introduced for the diagnosis and follow-up of glaucoma. In terms of diagnostic power and observer-independency, this technique seems to surpass the previous clinical standard of morphometric optic nerve head analysis, confocal scanning laser tomography (CSLT) measurement^1,3,4^. With this technique, retinal structures between Bruch’s membrane opening (BMO) and the internal limiting membrane (ILM) are evaluated, and sub-surface structures are analysed^1,5–12^. The correlations between morphometric ONH parameters and visual field function have been evaluated in several studies^1,13–23^.

The calculation of BMO-MRA values requires the identification of the smallest minimum rim area through which the nerve fibres pass between their retinal origin and the lamina cribrosa. To calculate the minimum surface between BMO and the ILM, Gardiner *et al*. used 48 trapezoids and minimized the area of each one independently. However, this simplification introduces discontinuities in the overall minimum rim surface between adjacent trapezoids. Therefore, this approach may not accurately reflect the true total minimum area through which the nerve fibres pass, which is determined by a continuous minimum surface around the ONH.

Here, we first describe a modified BMO-MRA calculation strategy that yields the continuous minimum rim surface within a triangular discretization that is globally optimized in all (typically 48) degrees of freedom simultaneously. We evaluate this novel tool in a large cohort of glaucoma, ocular hypertension and control patients compared to the standard clock-hour sector-wise sequential optimization technique. We aimed to verify the hypothesis that independent sequential optimization of clock-hour sector-wise BMO-MRA surfaces is not inferior to the simultaneously optimized continuous rim surface (BMO-gMRA) method.

## Methods

Data for this single-centre, retrospective analysis were acquired from 07/2014 to 03/2016 at the Department of Ophthalmology, University Hospital of Cologne, Germany. Patients received SD-OCT measurements of the optic nerve head. Additional parameters were collected from patients’ files: ophthalmologic diagnoses, previous surgery (especially if anti-glaucomatous), best-corrected visual acuity (BCVA), intraocular pressure (IOP) at examination and maximal IOP, topical medication, and medical history of the eyes. IOP was assessed by corneal rebound tonometry (Icare tonometer TA01i, Icare Finland Oy, Vantaa, Finland) and by Goldmann tonometry in adults and children, respectively. Individuals with no suspicion of glaucoma received ONH morphometry and visual field testing as part of a comprehensive screening for ocular diseases or for the exclusion of glaucoma or OHT, mainly on the basis of private medical practice.

All patients who received SD-OCT of the optic nerve head at the centre in the respective timeframe were eligible for inclusion in this study. The exclusion criteria included visual field loss due to aetiologies other than glaucoma and unsatisfactory image quality in SD-OCT or in visual field testing. If multiple data points at different time points were available, the examination with the best imaging quality indicators was used. The patients were classified into three diagnostic groups according to the 4^th^ Edition of Guidelines of the European Glaucoma Society (EGS, 2014)^24^: (1) glaucoma patients, (2) patients with ocular hypertension, and (3) individuals with no suspicion of glaucoma. Perimetric data were reviewed manually for glaucomatous changes according to the EGS guidelines^24^. Comprehensive grading and screening for glaucomatous defects in the visual field included evaluations of the mean deviation (MD), the mean pattern standard deviation (PSD), the greyscale map, the Bebié curve and pattern deviation probability maps. In cases of divergent diagnoses in both eyes of the same individual, the more unfavourable diagnosis was used to classify the patient. The aim of this approach was to reduce a potential selection bias for non-glaucomatous individuals recruited from the patients seen at our centre.

### Morphometric analyses of the optic disc

SD-OCT examinations were performed by *Spectralis®-*SD-OCT (Heidelberg Engineering GmbH, Heidelberg, Germany) according to standard operating and imaging procedures using a light source of 870 nm. The included OCT data had an image quality index of >15 dB. The scanning pattern was centred on the BMO with radial equidistance (24 high-resolution 15° radial scans, each averaged from 27 B-scans). The examiners controlled the centration of the scan to the optic disc and corrected errors in the detection of the internal limiting membrane (ILM) and Bruch’s membrane opening. OCT-based parameters were calculated by the device operating software tool provided by Heidelberg Engineering, including a source data export batch provided by the manufacturer.

**Figure 1:**
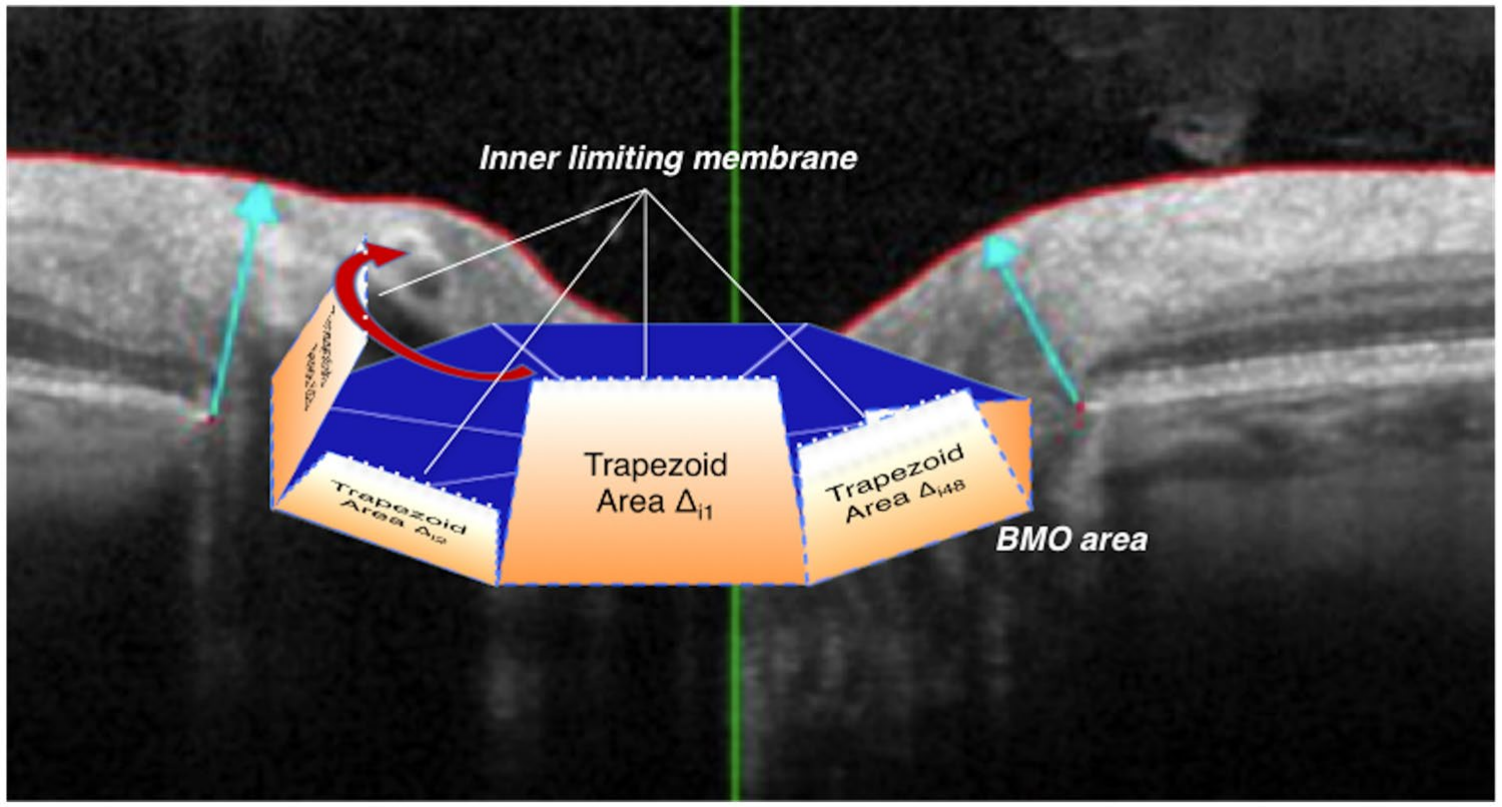
Principle of the BMO-MRA calculation in Spectralis SP-X VWM software. Figure 1a Graphical model; between every two B-scans, the local BMO-based minimum rim area is calculated as a trapezoid covering the minimum area between the internal limiting membrane (ILM) and Bruch**’**s membrane opening (BMO); Global and sectorial BMO-MRA values are determined by the addition of respective local minimum rim area results; model displays extreme tilt between two segments for purpose of illustration; BMO, Bruch**’**s membrane opening; BMO-MRA, BMO-based minimum rim area. (Note: graphical model modified from a version published previously^2^; (figure created with Microsoft Powerpoint of Microsoft Office Plus 2010, Microsoft Corporation).

BMO-MRA calculation was performed by Heidelberg Engineering software, *Spectralis SP-X VWM*. Gardiner and associates proposed the principle of the BMO-MRA calculation^1^. The BMO-MRA is described as the minimum surface between Bruch’s membrane opening and the ILM. The minimum surface tends to be approximately perpendicular to the direction of the nerve fibres traversing the optic nerve head. Therefore, the minimum surface tends to correlate with the total number of nerve fibres traversing the optic nerve. For BMO-MRA, similar to the procedures applied for the one-dimensional morphometric parameter MRW, the calculation software applied sequential minimization of every radial scan independently of all other sectors (Fig. 1).

**Figure 2:**
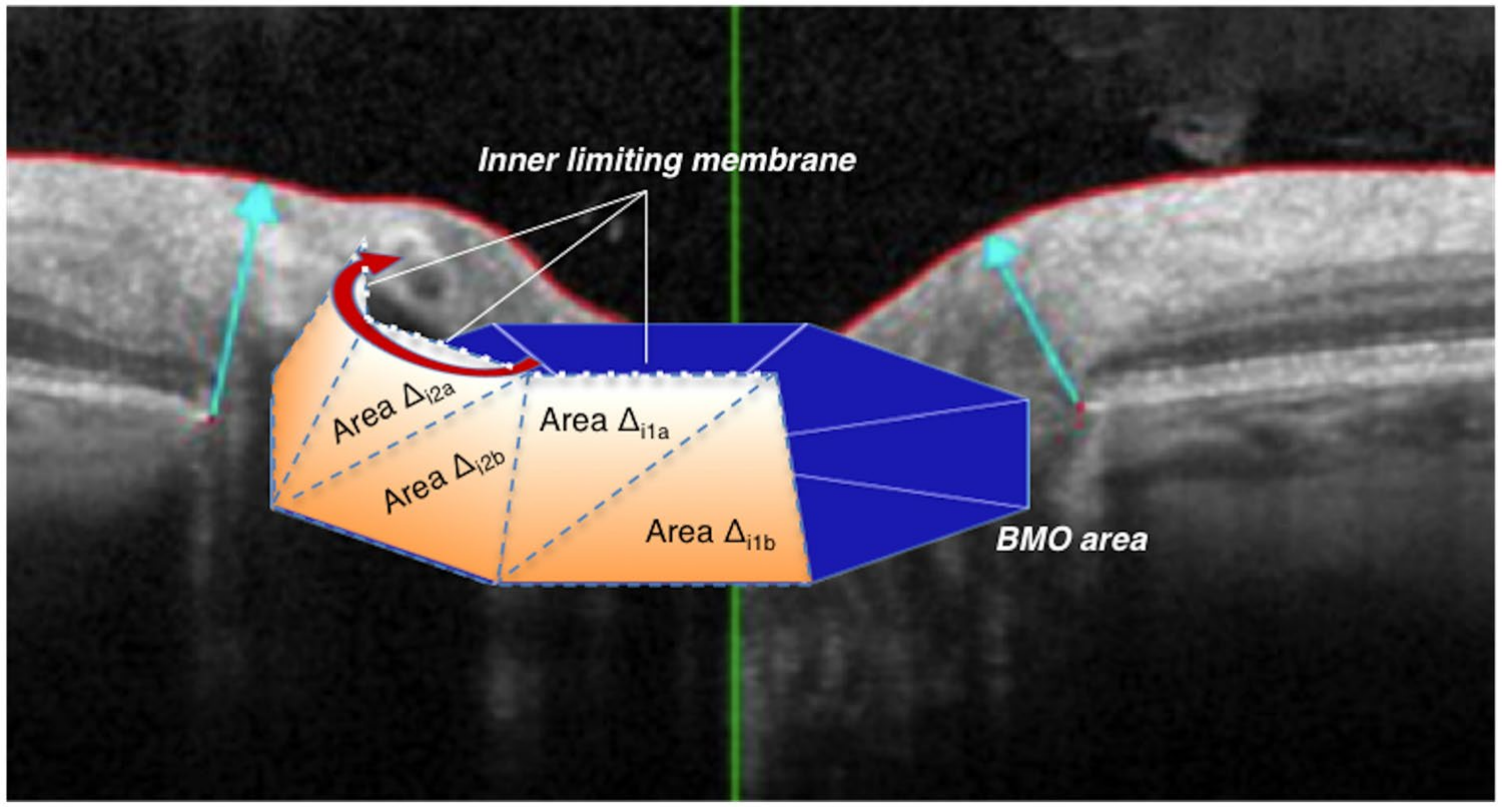
Principle the BMO-gMRA calculation in Spectralis SP-X VWM software. Graphical model; The gMRA surface is locally discretized in each SD-OCT star scan sector *i* as two adjacent triangles with areas ∆*i*1 and ∆*i*2. While BMO positions remain fixed during optimization, the position of BMO-ILM connections (i.e., the arrow in the OCT image) is optimized along the ILM segmentation line of the star. In this approach, global optimization is performed in all (typically 48) degrees of freedom simultaneously. BMO, Bruch’s membrane opening; BMO-gMRA, BMO-based globally optimized minimum rim area (Figure created with Microsoft Powerpoint of Microsoft Office Plus 2010, Microsoft Corporation).

Due to this sequential optimization strategy that does not include any constraints between neighbouring clock-hour sectors, discontinuities in the resulting minimum rim surface can arise between adjacent trapezoids if their vertical tilt angles differ. To determine the impact of such discontinuities on the resulting BMO-MRA parameter, Heidelberg Engineering provided an alternative computation of the continuous minimum rim surface (BMO-gMRA) within their *Spectralis SP-X software* for scientific use. Here, the BMO-gMRA is discretized by a sequence of (typically 96) triangles that span the area between BMO and the ILM. In each SD-OCT star scan of clock-hour sector *i*, two adjacent triangles with areas ∆*i*1 and ∆*i*2 span the surface locally (Fig. 2). While BMO positions remain fixed during optimization, the position of BMO-ILM connections (i.e., the arrow in the OCT image) is optimized along the ILM segmentation line of the star scan. In this way, all triangles are optimized simultaneously such that their total area (i.e., ∑(i)*Ai* = ∑(i)(∆*i*_1_ + ∆*i*_2_), where the sum runs over all OCT star scan sectors) is minimal under the additional constraint that the edges of adjacent triangles coincide. Within the limits of the triangular discretization, the BMO-gMRA algorithm provides the exact global minimum continuous surface between BMO and ILM segmentations of an SD-OCT star scan. As the orientation of the shared edge in pairs of triangles in each sector introduces an effective chirality in the discretization of the surface, this orientation is reversed when analysing opposite eyes (OD/OS) to avoid any bias.

### Ethics and statistics

According to national medical regulations on retrospective single-centre clinical studies, the Ethics Committee of the University of Cologne ruled that approval was not required for this study. All tenets of the declaration of Helsinki were observed. Statistical analyses were performed by SPSS (Version 22.0, IBM Corp. Armonk, NY, USA) and the statistical programming language R V3.2.2 (R Foundation for Statistical Computing, Vienna, Austria). Student’s T-Test or the Kolmogorov-Smirnov Z test was applied to compare means depending on normal distribution tested by the Shapiro-Wilk test. Diagnostic power was assessed by a receiver operating characteristic (ROC) analysis and tested for statistically significant differences with the DeLong test for areas under the curves. Bootstrap estimated p-values for the comparisons between partial area under the curves (pAUCs) of MRA and gMRA were reported. Benjamini-Hochberg correction was applied when testing for statistical significance to account for multiple testing. The resulting threshold for statistical significance was P < 0.011.

### Data availability statement

The datasets generated during and/or analysed during the current study are available from the corresponding author on reasonable request.

### Third party rights

Spectralis SP-X VWM software is exclusive property of Heidelberg Engineering GmbH, Heidelberg, Germany.

## Results

A total of 704 eyes of 445 patients were enrolled in this study, including 288 glaucoma patients (64.7%), 39 ocular hypertension patients (8.8%), and 118 healthy participants (26.5%). Epidemiology and baseline data are summarized in Table 1.

**Table 1:** Epidemiological and baseline data.

	Glaucoma (n = 449)	Normal controls (n = 189)	Ocular Hyper-tension (OHT) (n = 67)
**Gender n (%)**			
Men	198 (44.1)	87 (46.0)	33 (49.3)
Women	251 (55.9)	102 (54.0)	34 (50.7)
**Age (years)**			
Mean ± SD	64.5 ± 14.6	48.6 ± 19.8	53.1 ± 18.3
Median	67.0	51.0	54.0
**Eye n (%)**			
Right	224 (50.1)	93 (49.2)	36 (53.7)
Left	225 (49.9)	96 (50.8)	31 (46.3)
**BCVA in logMAR**			
Mean ± SD	0.13 ± 0.14	0.04 ± 0.10	0.04 ± 0.10
Median	0.1	0.0	0.0
**Refraction (Spherical equivalent) in diopters**			
Mean ± SD	−1.17 ± 2.07	−0.95 ± 1.91	−2.13 ± 3.04
Median	−0.61	−0.53	−1.34
**IOP (mmHg)**			
Mean ± SD	17.6 ± 6.1	14.8 ± 5.5	24.2 ± 6.9
Median	17.0	14.0	22.0
**Mean deviation (dB) of 30/2 visual field testing**			
Mean ± SD	−6.2 ± 6.5	−0.3 ± 1.4	0.9 ± 0.2
Median	−4.4	−0.3	1.0

Legend: SD, standard deviation; BCVA, best corrected visual acuity; IOP, intraocular pressure; dB, decibel; n/a, not applicable.

In all eyes, the mean BMO area was 1.89 ± 0.05 mm^2^, the mean global BMO-MRA was 0.97 ± 0.34 mm^2^, and the mean global BMO-gMRA was 1.01 ± 0.36 mm^2^. Both parameters correlated with r = 0.995 (P < 0.001) and showed a statistically significant mean difference of 0.04 mm^2^ (95% Confidence interval 0.03–0.04 mm^2^, P < 0.001). In all ONH sectors, BMO-MRA was statistically significantly smaller by 3.0–4.2% compared to gBMO-MRA (P < 0.001, respectively). Table 2 displays detailed data for all ONH sectors.

**Table 2:** Global and sectorial morphometric parameters in the ONH analysis.

		Global	Nasal	Nasal superior	Nasal inferior	Temporal	Temporal superior	Temporal inferior
**Full cohort**	BMO-MRA, mm^2^ ± SD	0.97 ± 0.3	0.32 ± 0.1	0.12 ± 0.0	0.13 ± 0.1	0.18 ± 0.1	0.10 ± 0.0	0.12 ± 0.1
	BMO-gMRA, mm^2^ ± SD	1.01 ± 0.4	0.33 ± 0.1	0.12 ± 0.0	0.14 ± 0.1	0.19 ± 0.1	0.11 ± 0.0	0.12 ± 0.1
**Glaucoma**	BMO-MRA, mm^2^ ± SD	0.82 ± 0.3	0.28 ± 0.1	0.10 ± 0.0	0.11 ± 0.0	0.16 ± 0.1	0.08 ± 0.0	0.09 ± 0.0
	BMO-gMRA, mm^2^ ± SD	0.86 ± 0.3	0.29 ± 0.1	0.11 ± 0.0	0.12 ± 0.0	0.17 ± 0.1	0.09 ± 0.0	0.10 ± 0.1
**Normal controls**	BMO-MRA, mm^2^ ± SD	1.24 ± 0.2	0.39 ± 0.1	0.15 ± 0.0	0.17 ± 0.0	0.23 ± 0.1	0.13 ± 0.0	0.16 ± 0.0
	BMO-gMRA, mm^2^ ± SD	1.25 ± 0.3	0.41 ± 0.1	0.16 ± 0.0	0.18 ± 0.0	0.24 ± 0.1	0.14 ± 0.0	0.17 ± 0.0
**Ocular Hypertension**	BMO-MRA, mm^2^ ± SD	1.21 ± 0.3	0.39 ± 0.1	0.15 ± 0.0	0.16 ± 0.0	0.23 ± 0.1	0.13 ± 0.0	0.15 ± 0.0
	BMO-gMRA, mm^2^ ± SD	1.26 ± 0.3	0.41 ± 0.1	0.16 ± 0.0	0.17 ± 0.0	0.23 ± 0.1	0.14 ± 0.0	0.15 ± 0.0

Legend: SD, standard deviation; ONH, optic nerve head; SD-OCT, spectral domain optical coherence tomography; BMO-MRA, Bruch’s membrane opening minimum rim area; BMO-gMRA, Bruch’s membrane opening globally optimized minimum rim area.

These differences were stable between diagnostic groups. In healthy controls, global BMO-MRA was smaller on average by 0.04 mm^2^ compared to BMO-gMRA (P < 0.001). In glaucoma patients, the difference between global BMO-gMRA and global BMO-MRA was 0.03 mm^2^ (P < 0.001). In eyes with moderate to severe damage to visual field function, reflected by an MD inferior to −6.0 dB, BMO-MRA was smaller on average by 0.025 mm^2^ compared to BMO-gMRA (P < 0.001).

In glaucoma patients, the correlation with MD in visual field testing showed a slightly higher correlation for BMO-MRA, with rho (ρ) = 0.68, compared to BMO-gMRA with ρ = 0.66. This difference was statistically significant (P < 0.001).

The receiver operating characteristic (ROC) analysis was performed to assess and compare the diagnostic powers of the two analysed morphometric parameters. The area under the curve (AUC) was 0.873 for global BMO-MRA and 0.866 for global BMO-gMRA (P = 0.004). Sensitivities at 90%-specificity were at 73.3% and 73.6%, respectively. For ONH sectors, the AUC was highest for temporal inferior BMO-MRA at 0.88.

**Figure 3:**
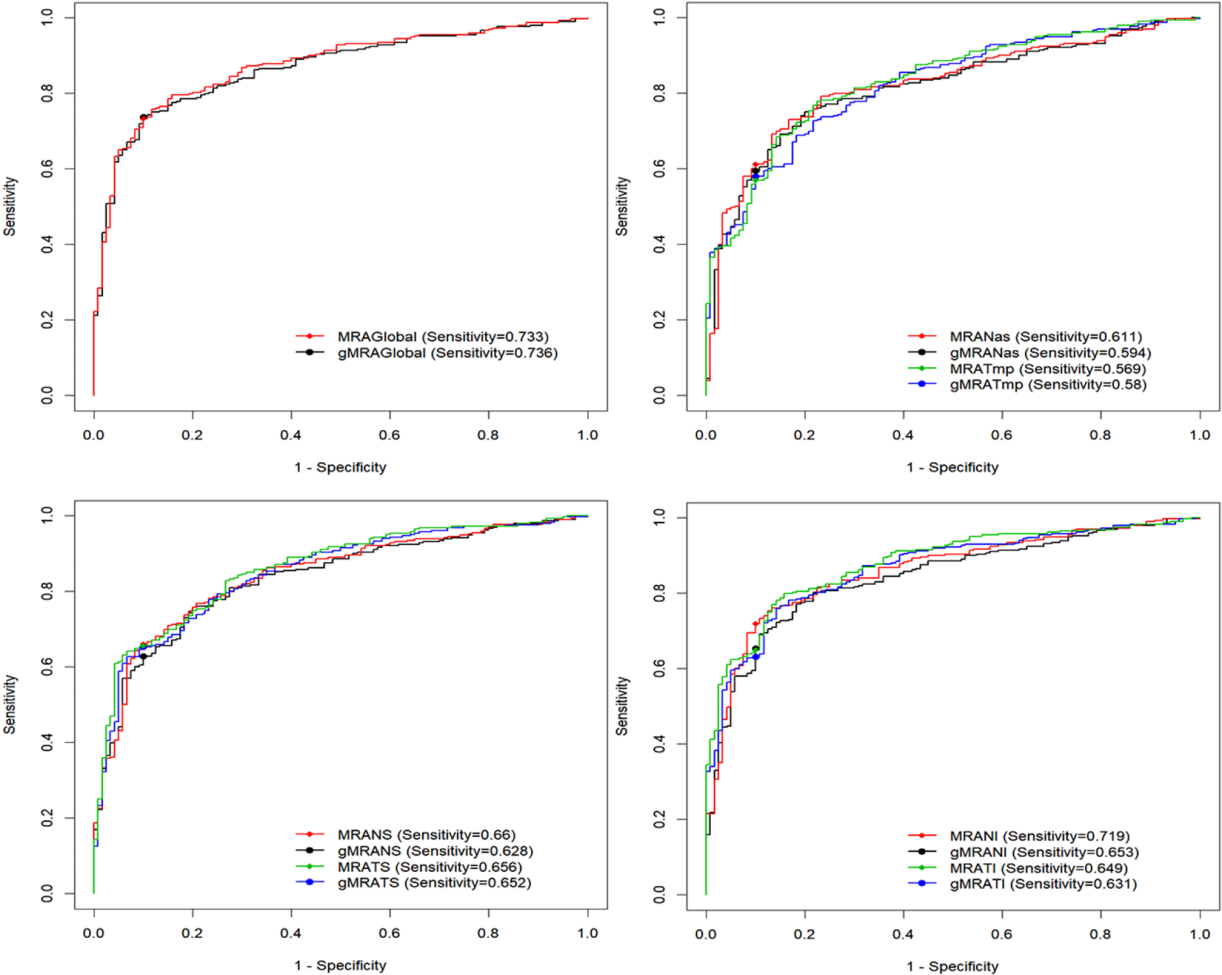
ROC analysis of BMO-MRA and BMO-gMRA for the discrimination of glaucoma patients from normal controls with sensitivities at 90% specificity. • Top-right: Global. • Top-left: Central nasal and temporal sectors. • Bottom-right: Inferior nasal and temporal sectors. • Bottom-left: Superior nasal and temporal sectors. ROC, Receiver Operator Characteristic; BMO-MRA, Bruch’s membrane opening-based minimum rim area; BMO-MRW, Bruch’s membrane opening-based minimum rim width; RNFL, Retinal nerve fibre layer; DM-RA, disc margin rim area (Figure created with R V3.2.2, R Foundation for Statistical Computing, Vienna, Austria).

Glaucoma patients and controls differed significantly in ONH size (1.97 ± 0.49 mm²; 2.36 ± 0.67; P < 0.001) and in mean age (60.88 ± 23.8 years; 43.88 ± 22.9; P < 0.001). To exclude a potential bias caused by these differences, the ROC analyses were repeated for 230 eyes of 159 patients in both groups aged 55 years or older and with an ONH size in CSLT of 1.63 to 2.43 mm², corresponding to the normal range of ONH size in CSLT software. While the AUCs of the morphometric parameters were slightly decreased by approximately 0.04 on average, the differences in AUCs between optimization approaches and between ONH sectors were constant (Tables 3 and 4). Figure 3 shows the results of the ROC analyses.

**Table 3:** ROC Analysis for sensitivity assessment of the morphometric parameters at 95% and 90% specificity (full cohort).

Glaucoma patients versus Controls							
		Sensitivity at 95%-specificity	Sensitivity at 90%-specificity	AUC	AUC Confidence Interval*	pAUC^†^	pAUC Confidence Interval*
**Global**	BMO-MRA	64.9%	73.3%	0.873	0.84–0.91	0.055	0.04–0.07
	BMO-gMRA	63.5%	73.6%	0.866	0.83–0.90	0.055	0.04–0.07
**Nasal**	BMO-MRA	49.7%	61.1%	0.821	0.78–0.86	0.042	0.03–0.06
	BMO-gMRA	44.8%	59.4%	0.814	0.77–0.86	0.041	0.03–0.06
**Nasal superior**	BMO-MRA	43.1%	66.0%	0.841	0.80–0.88	0.044	0.03–0.06
	BMO-gMRA	44.1%	62.8%	0.833	0.79–0.87	0.044	0.03–0.05
**Nasal inferior**	BMO-MRA	58.3%	71.9%	0.860	0.82–0.90	0.05	0.034–0.07
	BMO-gMRA	53.8%	65.3%	0.842	0.80–0.88	0.05	0.033–0.06
**Temporal**	BMO-MRA	41.7%	56.9%	0.832	0.79–0.87	0.04	0.034–0.05
	BMO-gMRA	44.4%	58.0%	0.821	0.78–0.86	0.04	0.034–0.05
**Temporal superior**	BMO-MRA	61.3%	65.6%	0.859	0.82–0.90	0.05	0.037–0.06
	BMO-gMRA	58.8%	65.2%	0.848	0.81–0.89	0.05	0.03–0.06
**Temporal inferior**	BMO-MRA	62.4%	64.9%	0.879	0.85–0.91	0.06	0.05–0.07
	BMO-gMRA	59.6%	63.1%	0.866	0.83–0.90	0.05	0.04–0.06

Note: *Asymptotic 95% Confidence Interval. ^†^Partial AUCs (pAUC) for specificity of 1–0.9;

Legend: AUC, Area under curve; Note: ROC, Receiver operator characteristic; BMO-MRA, Bruch’s membrane opening minimum rim area; BMO-gMRA, Bruch’s membrane opening globally optimized minimum rim area.

**Table 4:** DeLong-Test and bootstrap estimated p-values to compare AUCs and pAUCs of morphometric parameters.

	AUC of BMO-MRA	AUC of BMO-gMRA	P Value	pAUC of BMO-MRA	pAUC of BMO-gMRA	P Value
Global	0.873	0.866	0.004	0.055	0.055	0.94
Nasal	0.821	0.814	0.221	0.042	0.041	0.744
Nasal superior	0.841	0.833	0.131	0.044	0.044	0.876
Nasal inferior	0.860	0.842	<0.001	0.049	0.046	0.178
Temporal	0.832	0.821	0.05	0.041	0.042	0.53
Temporal superior	0.859	0.848	0.029	0.051	0.047	0.048
Temporal inferior	0.879	0.866	0.006	0.056	0.052	0.154

Legend: AUC, Area under curve; ROC, Receiver operator characteristic; partial AUCs (pAUC) for specificity of 1–0.9; BMO-MRA, Bruch’s membrane opening minimum rim area; BMO-gMRA, Bruch’s membrane opening globally optimized minimum rim area.

## Discussion

In recent studies on Bruch’s membrane opening minimum rim area in SD-OCT, the morphometric parameter was calculated using an algorithm with localized minimization of 48 independent trapezoids^1,2^. Whether this approach reflects the best the minimum surface between Bruch’s membrane opening and ILM was unclear. The minimum surface tends to be approximately perpendicular to the direction of the nerve fibres traversing the optic nerve head. In the previously used algorithm, neighbouring independently minimized trapezoids could show significant relative tilts because each trapezoid was optimized without any continuity constraint with respect to its adjacent neighbours.

An alternative algorithm using simultaneous optimization of the area in all clock-hour sectors under additional continuity constraints to minimize the BMO-based minimum rim area (BMO-gMRA) was provided within the scientific Spectralis SPX-VWM software by Heidelberg Engineering.

In this study, we compared and evaluated both optimization strategies for the BMO-based minimum rim area. BMO-MRA showed smaller values for the ONH globally and in all individual sectors compared to BMO-gMRA. This observation can be rationalized as BMO-gMRA optimization being subject to additional continuity constraints on the resulting surface, which limits the possible minimum solutions to a smaller subset (i.e., any surface that is smaller but discontinuous is not a valid optimum solution anymore).

In a direct comparison, BMO-MRA showed a slightly better correlation to visual field function in 30:2 white on white perimetry. Furthermore, the diagnostic power to differentiate glaucoma patients from healthy controls was comparable.

With the increasing use of BMO-based SD-OCT in glaucoma diagnostics, the performances of the different morphometric parameters to differentiate glaucomatous damage are analysed and challenged for different ONH characteristics^9–11^. The BMO-based minimum rim area analysis has been proposed as the next step in advancing the technique^1,13^. Generally, BMO-based minimum rim area parameters have been shown to offer comparability in the assessment of neuro-retinal rim tissue between large differences in ONH size.

The limitations of our study include the retrospective design and a potential selection bias due to patient exclusion because of data inconsistencies or insufficient image quality. The relatively large sample size in the present study reduces this possible bias.

To summarize the main conclusions of the present study, the simultaneous global optimization of the BMO-based minimum rim area shows that the imposition of an adjacency constraint for trapezoid area calculations is not superior compared to the established approach of independent sequential minimization of the individual areas in each clock-hour sector. Therefore, the results of previous studies on BMO-MRA can be regarded as valid. It does not seem necessary to adjust the established strategy for minimum rim area optimization in the clinical routine for glaucoma diagnostics.

## References

[1] Gardiner SK (2014). A method to estimate the amount of neuroretinal rim tissue in glaucoma: comparison with current methods for measuring rim area. Am J Ophthalmol.

[2] Enders P (2016). Novel Bruch’s Membrane Opening Minimum Rim Area Equalizes Disc Size Dependency and Offers High Diagnostic Power for Glaucoma. Invest Ophthalmol Vis Sci.

[3] Reis AS (2012). Influence of clinically invisible, but optical coherence tomography detected, optic disc margin anatomy on neuroretinal rim evaluation. Invest Ophthalmol Vis Sci.

[4] Reis AS (2012). Optic disc margin anatomy in patients with glaucoma and normal controls with spectral domain optical coherence tomography. Ophthalmology.

[5] Povazay B (2007). Minimum distance mapping using three-dimensional optical coherence tomography for glaucoma diagnosis. J Biomed Opt.

[6] Abramoff MD (2009). Automated segmentation of the cup and rim from spectral domain OCT of the optic nerve head. Invest Ophthalmol Vis Sci.

[7] Mwanza JC, Oakley JD, Budenz DL, Anderson DR (2011). & Cirrus Optical Coherence Tomography Normative Database Study, G. Ability of cirrus HD-OCT optic nerve head parameters to discriminate normal from glaucomatous eyes. Ophthalmology.

[8] Chen TC (2009). Spectral domain optical coherence tomography in glaucoma: qualitative and quantitative analysis of the optic nerve head and retinal nerve fiber layer (an AOS thesis). Trans Am Ophthalmol Soc.

[9] Chauhan BC, Burgoyne CF (2013). From clinical examination of the optic disc to clinical assessment of the optic nerve head: a paradigm change. Am J Ophthalmol.

[10] Chauhan BC (2015). Bruch’s Membrane Opening Minimum Rim Width and Retinal Nerve Fiber Layer Thickness in a Normal White Population: A Multicenter Study. Ophthalmology.

[11] Chauhan BC (2013). Enhanced detection of open-angle glaucoma with an anatomically accurate optical coherence tomography-derived neuroretinal rim parameter. Ophthalmology.

[12] Bowd C (2006). Structure-function relationships using confocal scanning laser ophthalmoscopy, optical coherence tomography, and scanning laser polarimetry. Invest Ophthalmol Vis Sci.

[13] Muth DR, Hirneiss CW (2015). Structure-Function Relationship Between Bruch’s Membrane Opening-Based Optic Nerve Head Parameters and Visual Field Defects in Glaucoma. Invest Ophthalmol Vis Sci.

[14] Gardiner SK, Johnson CA, Cioffi GA (2005). Evaluation of the structure-function relationship in glaucoma. Invest Ophthalmol Vis Sci.

[15] Anton A, Yamagishi N, Zangwill L, Sample PA, Weinreb RN (1998). Mapping structural to functional damage in glaucoma with standard automated perimetry and confocal scanning laser ophthalmoscopy. Am J Ophthalmol.

[16] Anderson RS (2006). The psychophysics of glaucoma: improving the structure/function relationship. Prog Retin Eye Res.

[17] Caprioli J (1989). Correlation of visual function with optic nerve and nerve fiber layer structure in glaucoma. Surv Ophthalmol.

[18] Garway-Heath DF, Holder GE, Fitzke FW, Hitchings RA (2002). Relationship between electrophysiological, psychophysical, and anatomical measurements in glaucoma. Invest Ophthalmol Vis Sci.

[19] Harwerth RS, Carter-Dawson L, Smith EL, Crawford ML (2005). Scaling the structure–function relationship for clinical perimetry. Acta Ophthalmol Scand.

[20] Enders, P., Schaub, F., Hermann, M. M., Cursiefen, C. & Heindl, L. M. Neuroretinal rim in non-glaucomatous large optic nerve heads: a comparison of confocal scanning laser tomography and spectral domain optical coherence tomography. *Br J Ophthalmol*, doi:10.1136/bjophthalmol-2015-307730 (2016). 10.1136/bjophthalmol-2015-307730 27118190

[21] Schlottmann PG, De Cilla S, Greenfield DS, Caprioli J, Garway-Heath DF (2004). Relationship between visual field sensitivity and retinal nerve fiber layer thickness as measured by scanning laser polarimetry. Invest Ophthalmol Vis Sci.

[22] Enders, P. *et al*. The use of Bruch’s membrane opening-based optical coherence tomography of the optic nerve head for glaucoma detection in microdiscs. *Br J Ophthalmol*, 10.1136/bjophthalmol-2016-308957 (2016). 10.1136/bjophthalmol-2016-308957 27436783

[23] Enders P, Schaub F, Heindl LM (2017). Spectral-Domain Optical Coherence Tomography-Derived Characteristics of Bruch Membrane Opening in a Young Adult Australian Population. Am J Ophthalmol.

[24] European Glaucoma Society. *Terminology and Guidelines for Glaucoma*. 4th edition edn, (PubliComm, 2014). 10.1136/bjophthalmol-2021-egsguidelines 34675001

